# Understanding a Nonlinear Causal Relationship Between Rewards and Physicians’ Contributions in Online Health Care Communities: Longitudinal Study

**DOI:** 10.2196/jmir.9082

**Published:** 2017-12-21

**Authors:** Jying-Nan Wang, Ya-Ling Chiu, Haiyan Yu, Yuan-Teng Hsu

**Affiliations:** ^1^ Key Laboratory of Electronic Commerce and Logistics School of Economics and Management Chongqing University of Posts and Telecommunications Chongqing China; ^2^ Health Big Data Research Institute Big Data Research Center University of Electronic Science and Technology of China Chengdu China; ^3^ Department of Statistics Eberly College of Science The Pennsylvania State University University Park, PA United States; ^4^ Research Center of Finance Shanghai Business School Shanghai China

**Keywords:** online health care community, physician online contribution, psychological reward, material reward

## Abstract

**Background:**

The online health care community is not just a place for the public to share physician reviews or medical knowledge, but also a physician-patient communication platform. The medical resources of developing countries are relatively inadequate, and the online health care community is a potential solution to alleviate the phenomenon of long hospital queues and the lack of medical resources in rural areas. However, the success of the online health care community depends on online contributions by physicians.

**Objective:**

The aim of this study is to examine the effect of incentive mechanisms on physician’s online contribution behavior in the online health community. We addressed the following questions: (1) from which specialty area are physicians more likely to participate in online health care community activities, (2) what are the factors affecting physician online contributions, and (3) do incentive mechanisms, including psychological and material rewards, result in differences of physician online contributions?

**Methods:**

We designed a longitudinal study involving a data sample in three waves. All data were collected from the Good Doctor website, which is the largest online health care community in China. We first used descriptive statistics to investigate the physician online contribution behavior in its entirety. Then multiple linear and quadratic regression models were applied to verify the causal relationship between rewards and physician online contribution.

**Results:**

Our sample included 40,300 physicians from 3607 different hospitals, 10 different major specialty areas, and 31 different provinces or municipalities. Based on the multiple quadratic regression model, we found that the coefficients of the control variables, past physician online contributions, doctor review rating, clinic title, hospital level, and city level, were .415, .189, –.099, –.106, and –.143, respectively. For the psychological (or material) rewards, the standardized coefficient of the main effect was 0.261 (or 0.688) and the standardized coefficient of the quadratic effect was –0.015 (or –0.049). All estimates were statistically significant (*P*<.001).

**Conclusions:**

Physicians with more past physician online contribution, with higher review ratings, coming from lower level clinics, not coming from tertiary hospitals, and not coming from big cities were more willing to participate in online health care community activities. To promote physician online contribution, it is necessary to establish an appropriate incentive mechanism including psychological and material rewards. Finally, our findings suggest two guidelines for designing a useful incentive mechanism to facilitate physician online contribution. First, material reward is more useful than psychological reward. Second, as indicated by the concave-down-increasing causal relationship between rewards and physician online contribution, although an appropriate reward is effective in encouraging willingness on the part of physicians to contribute to the online health care community, the effect of additional rewards is limited.

## Introduction

### Background

With the development of a mature online health care community, more and more people have begun to use online reviews within the online health care community to obtain information about the quality of their physicians [1]. This phenomenon has received the attention of many researchers, and several studies of the online health care community have been conducted focusing on various issues, such as how online physician reviews have been used in different countries [2-7], what the differences between the traditional and online physician reviews are [8], and whether differences in medical specialty areas affect these reviews [5,9]. Nevertheless, in China, even though the online health care community might help consumers look for a good physician, the queues at Chinese hospitals are legendary [10], meaning it is not easy to make an appointment with a physician. The reason is that medical resources are relatively insufficient in the country. Statistically, health spending accounts for only 5.5% of the gross domestic product, and there are approximately 1.8 doctors and 2.4 nurses for every 1000 people [11]. In China, health resources are far less than those in the Organisation for Economic Co-operation and Development countries. More importantly, substantial inequalities remain in the geographical distribution of medical resources; in particular, provinces in western China have the lowest levels of resources [12]. With its potential to mitigate the problems of the long waiting times at hospitals and the low levels of medical resources in rural areas, the online health care community is no longer merely a site for the public to share physician reviews; it has also become a physician-patient communication platform in China.

There have been many companies offering this type of service, among which the Good Doctor website is a typical example [5]. The Good Doctor website (*hao dai fu* means “good doctor” in Chinese) was the earliest online physician review website [13] and has been in operation in China since 2006. In 2016, it began working with the Yinchuan Municipal People’s Government and it has obtained a medical institution license so that it can provide new online medical services in China. According to the Good Doctor website, it included references to 7216 hospitals and more than 480,000 physicians at the end of 2016. Among these, approximately 142,000 physicians included their actual verified identities. They can directly provide medical advice to patients, make appointments for treatment, and share their professional knowledge. Of course, this online health care service cannot replace offline medical interaction entirely, but it can reduce the huge pressure on China’s health care system. The key factor determining the online health care community’s success is whether the physicians are actively involved in the sites. Therefore, understanding and promoting physician online contribution is a critical issue for the online health care community managers.

### Research Problem

Many studies have investigated online contribution behavior in other kinds of online communities such as Wikipedia [14,15], social Q&A sites [16-18], and open source software communities [19,20].The importance of member contribution for the sustainable development of online communities has been verified by extensive research [16-18,21]. Establishing an effective incentive mechanism is one of the most common ways to maintain community contribution behavior [22-26]. These related studies can be divided into three categories according to their research methods. In the first, the questionnaire survey is adopted to investigate the knowledge-sharing community [22-23].This type of study considers both extrinsic and intrinsic incentives and examines their effects on the member’s contribution behavior. The empirical results verify the significant positive effect of intrinsic incentives, but the influence of extrinsic incentives is inconsistent. Second, some studies have discussed the Q&A community [24] and the online learning community [25] by means of an experimental design. They consider only the effect of extrinsic incentive on contribution behavior, and they conclude that the extrinsic incentive has a significant positive effect on users’ online contributions. Third, applying Web technology to collect online community public data is another way to investigate this issue; researchers such as Raban [26] has explored members’ contributions in Google Answers, an online community to help users find expert information possessed by others online. A high-quality answer is scored at a higher rating and, as a way of expressing thanks, some askers might be willing to provide a tip, in the form of a voluntary gratuity payment. Thus, in the work of Raban, ratings and tips were used to measure intangible and tangible incentives, respectively. In addition, the number of answers was regarded as a proxy for a user’s contribution level. The empirical evidence indicates that both intangible and tangible incentives have a significant positive influence on users’ online contribution. Despite the fact that the online health care community has been in existence around the world for over a decade, very little is known about incentive mechanisms that could foster physicians’ willingness to contribute and interact with patients in the online health care community. The online health care community managers can establish incentive mechanisms, such as thank you letters and virtual gifts, which might encourage physician contributions. This study attempts to bridge this gap in our knowledge. We designed a longitudinal study to examine whether physician online contribution is affected by incentive mechanisms.

## Methods

### Research Model

**Figure 1:**
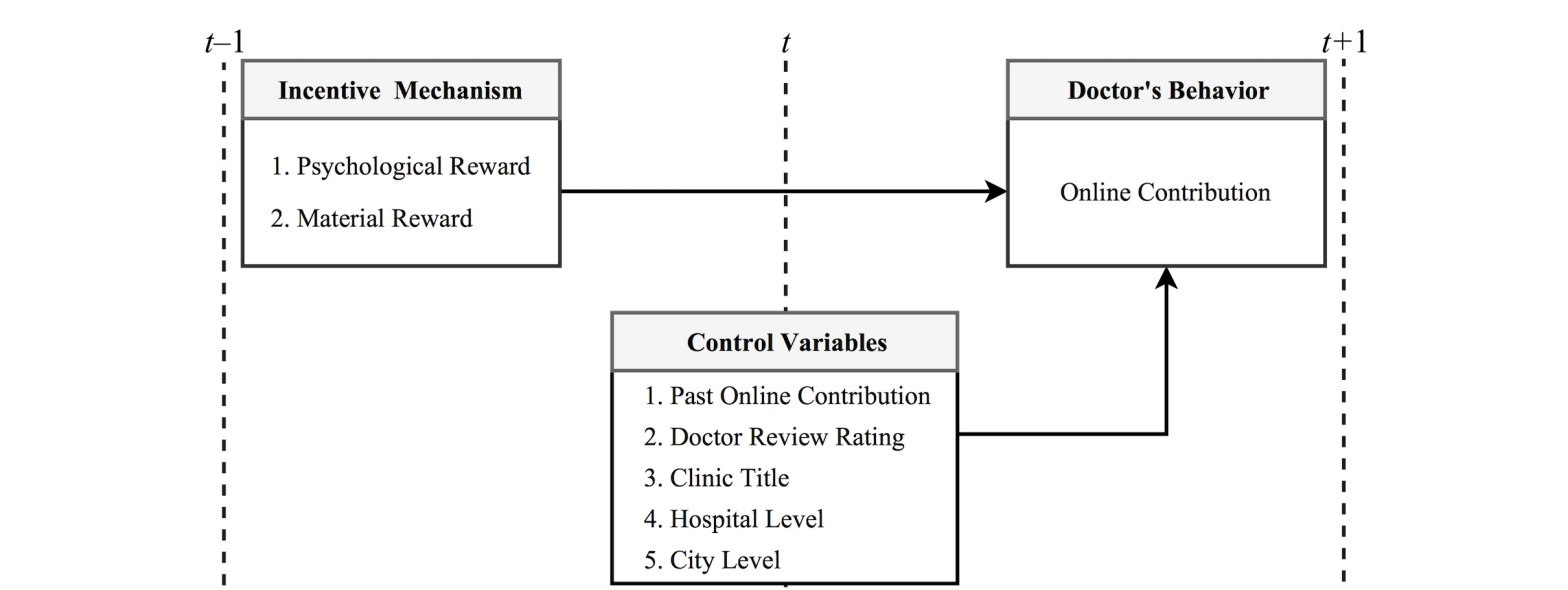
Summary of the proposed model of the effects of psychological and material rewards on physician online contribution. Rewards are measured from time t–1 to t. Online contribution is measured from time t to t+1. Control variables are measured at time t.

Figure 1 represents the research model. Five control variables—past physician online contribution, doctor review rating, clinic title, hospital level, and city level—represent the physician’s status at a specific time. In other words, these are all stock variables measured at time *t*. Psychological (intrinsic) and material (extrinsic) rewards are considered within the incentive mechanism [22,23,26]. Both of these are flow variables measured from time *t* –1 to *t*. Finally, the physician online contribution is also a flow variable, measured from time *t* to *t* +1. Based on this framework, we can verify whether physicians receiving certain rewards changes their online contribution behavior in the next period.

Reinforcing theory, which suggests that stimulus is used to shape behaviors [27], provides a relevant foundation to address the causal relationship between rewards and physician online contribution. From the view of social psychology, people’s attitude can be strengthened through intrinsic and extrinsic rewards, whether the effect is a reinforcing or a changing of attitude [28]. In other words, reward is a key factor in behavioral decisions [24,29,30] and can cause repetitive behaviors [27,31]. In this study, our incentive mechanism is comprised of psychological and material rewards. The psychological reward is measured as the number of thank you letters from patients [32]. This reward is regarded as a kind of intrinsic reward, enhancing physicians’ self-efficacy and self-worth [22,23,33]. The material reward is measured as the number of received token gifts, which are sold in the online health care community and are used to express gratitude to the physicians. These “virtual gifts” can be converted into a cash equivalent and then deposited into the physician’s personal research fund. Thus, they are a kind of extrinsic or economic benefit [22,23]. Further, in light of “the law of diminishing marginal utility” [34], a classic law in economics, we further explore how psychological and material rewards affect the physicians’ online contributions. Based on universal human experience, this law states that the marginal utility derived from each additional unit diminishes compared to that of the previous unit. In our context, when a physician receives more psychological or material rewards, there is a decline in the marginal effect of each additional reward on the physician’s online contribution. Mathematically, a function with a positive first derivative and a negative second derivative is termed a concave-down-increasing function. We investigate the existence of this concave-down-increasing relationship between rewards and physician online contribution.

### Data Collection and Processing

**Figure 2:**
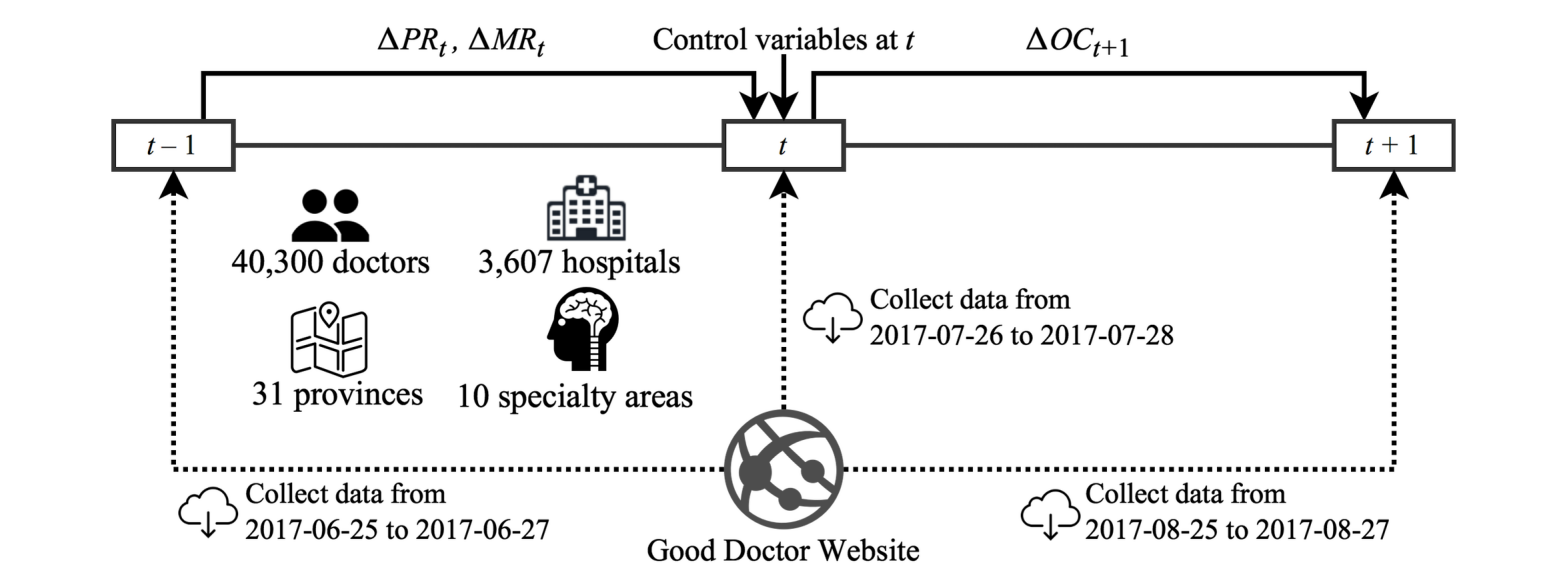
Data collection and processing. ∆PR_t_ and ∆MR_t_ represent the increment of psychological reward and material reward from time t–1 to t, respectively, and ∆OC_t+1_ represents the increment of physician online contribution from time t to t+1.

By means of Web crawler technology, data for this study were collected from the Good Doctor website on which more than 423,916 physicians’ profiles could be found. However, only after a physician applies for a personal webpage is he or she able to provide full online services (eg, online dialog with patients or the sharing of professional articles). Thus, the 142,457 doctors with personal webpages on the site were considered for the purposes of the study to be genuinely involved in the website, and others were not included in our sample. Further, to ensure that the doctor was currently active on the website, the most recent log-in time had to be within 1 month. Thus, we focused on 40,300 doctors who had personal websites and had logged into the Good Doctor website recently. To investigate whether adding psychological and material rewards would cause the physicians’ online contribution behavior to change, we designed a longitudinal study involving a data sample in three waves. The data collection process is shown in Figure 2. Specifically, at the start (June 25, 2017), we collected data including the physician’s ID and the numbers of received thank you letters and token gifts as proxies for psychological and material rewards. In a follow-up phase 1 month later (July 26, 2017), we collected a second wave of data including the doctor review ratings, clinic title, hospital level, city level, thank you letters, token gifts, and online contribution score. In the last phase (August 25, 2017), we collected each physician’s online contribution score again, covering the period of 1 month. It should be noted that due to the specific data collecting period, a seasonal bias may exist in our analysis (eg, many people, including physicians, usually have more vacations in summer).

### Sample Characteristics

**Figure 3:**
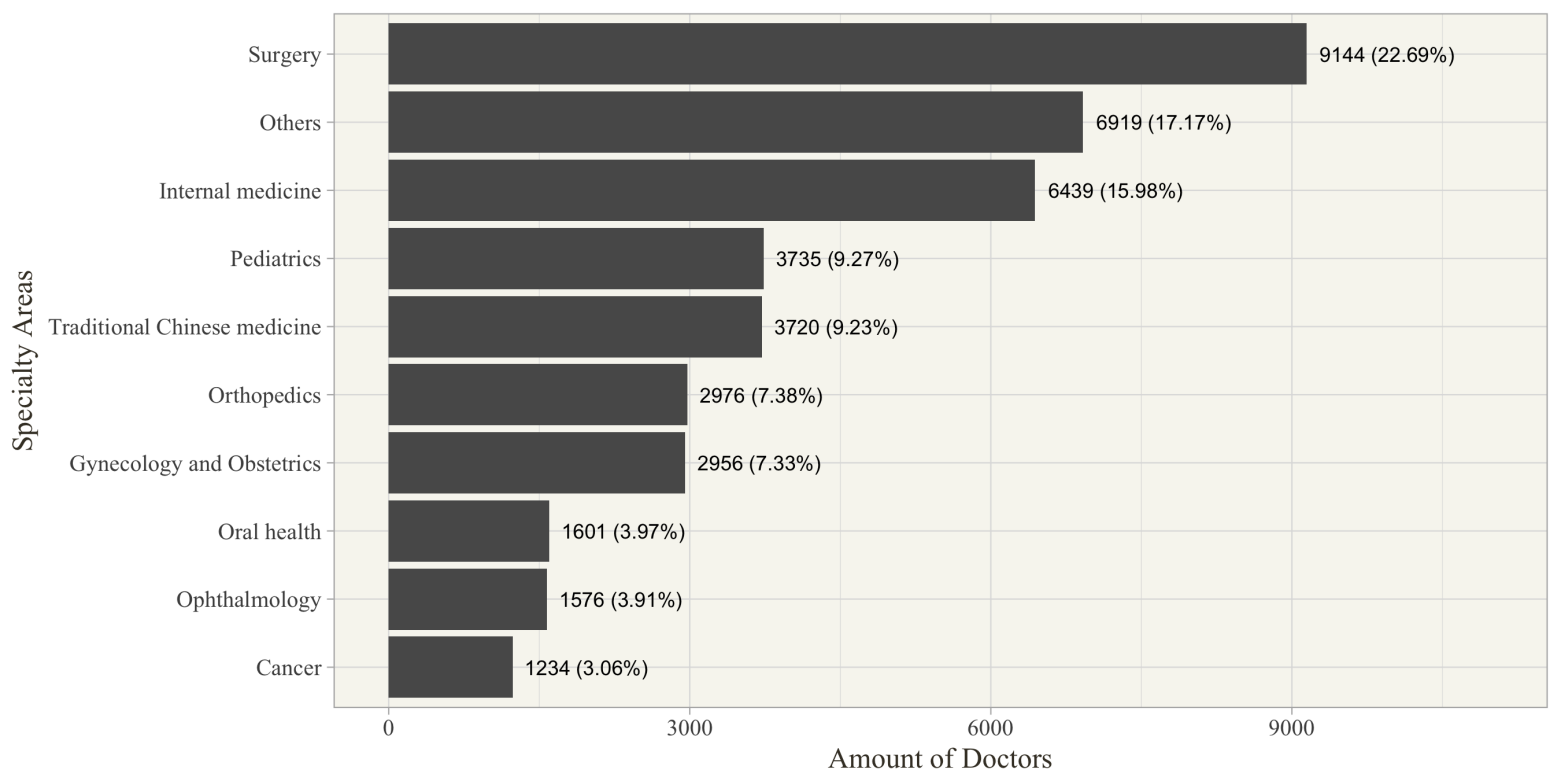
Number of doctors in 10 major specialty areas (N=40,300).

**Figure 4:**
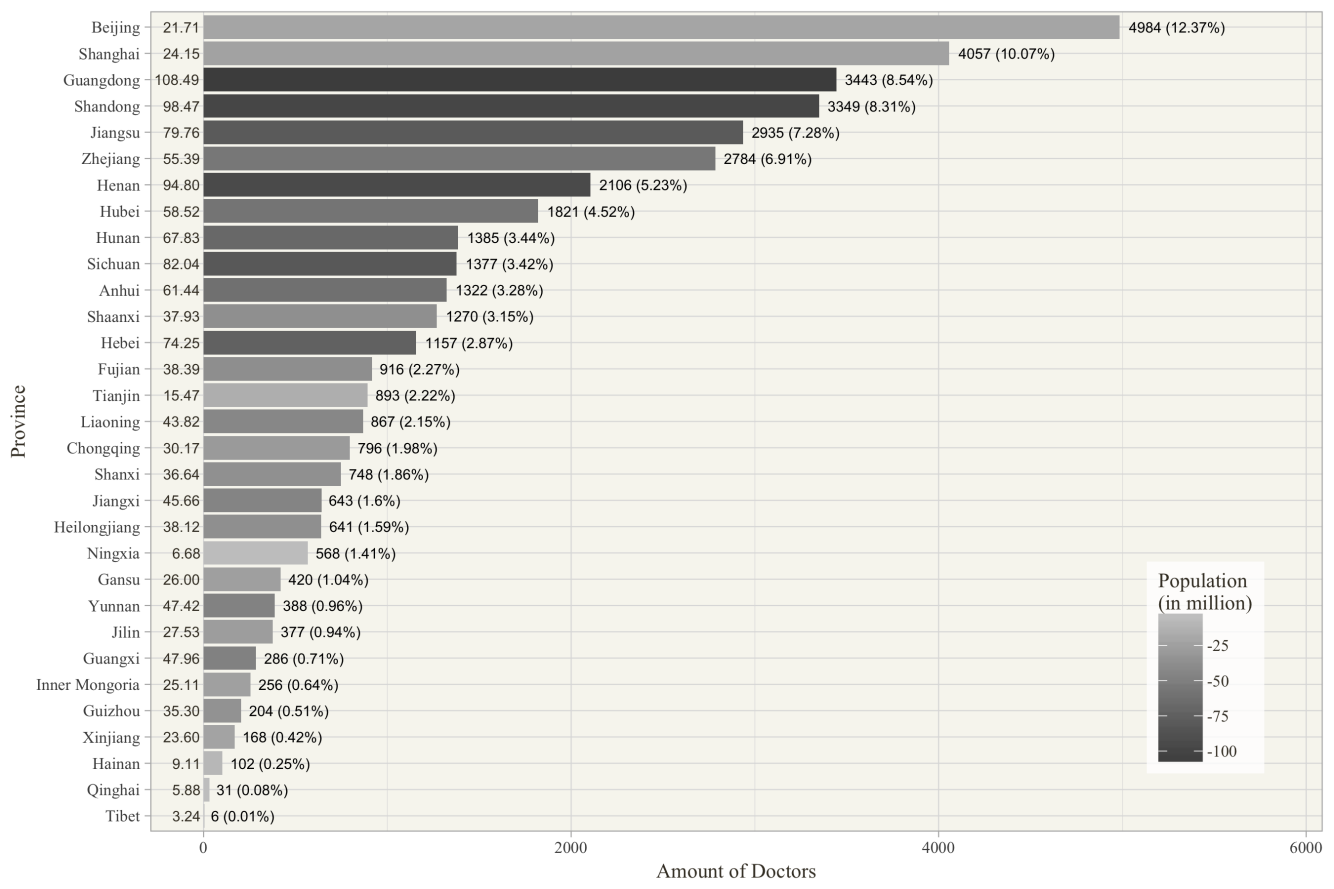
Number of doctors in 31 provinces or municipalities (N=40,300).

Some of the sample characteristics were worth additional exploration. First, the 40,300 doctors came from 3607 different hospitals, 10 different major specialty areas, and 31 different provinces or municipalities in China. This indicates that our sample was not confined to a specific group. In particular, Figure 3 shows the numbers of physicians in different major specialty areas. Surgery, internal medicine, pediatrics, and traditional Chinese medicine accounted for the largest numbers of physicians, with approximately 23%, 16%, 9%, and 9% of the total number of physicians, respectively. Figure 4 represents the numbers of physicians in 31 provinces or municipalities. In addition, Figure 4 also shows the corresponding populations in 2015, which can be generated from the National Bureau of Statistics of China [35]. In general, the larger population size comes with a larger number of physicians on the Good Doctor website, except for two big cities, Beijing and Shanghai, which are the China’s political and economic centers, respectively. Although the total population of permanent residents in Beijing and Shanghai accounts for only 3.3% of the total in China, approximately 22% of the physicians came from both cities. This might reflect the relative adequacy of medical resources in large cities or partially be due to the promotion strategies of the Good Doctor website. This naturally reflects the relative adequacy of medical resources in large cities. Second, the clinic title is unified nationally corresponding to four levels: resident physician, attending physician, associate chief physician, and chief physician (from junior to senior). These four levels account for 9.1%, 30.5%, 33.3%, and 27.1% of the total doctor population in our sample, respectively. Third, approximately 82% of the physicians come from hospitals in the tertiary category, which is the official certification of the highest quality hospitals. Finally, we also collected the doctors’ review ratings, which may be regarded as online word-of-mouth. The mean of these ratings was 3.84 (standard deviation [SD] 0.34) on a scale from 1 to 5, with 5 being the highest score.

### Measures

#### Online Contribution

**Figure 5:**
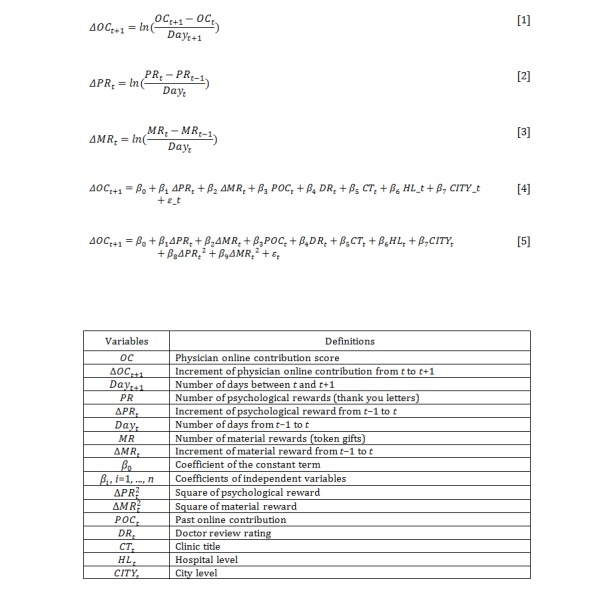
Equations and variable definitions.

Essentially, the existence of online contributions means that members are involved in community-related activities, such as sharing information actively, responding positively to other members’ questions, and intuitively interacting with other members [16,21]. In this study, we measured the physicians’ online contribution through the contribution scores listed on the Good Doctor website. There are three principle ways in which the contribution score can change. First, when physicians update their personal information, such as outpatient information and consultation range, in a timely manner, their contribution scores can be increased through the online health care community administrator’s audit. Second, physicians are encouraged to post medical articles for patients on the website. After the article is referenced by the Good Doctor website, the contribution score is updated. Third, if a physician can answer a patient’s question online, his or her contribution score will be increased. In this study, increment of physician online contribution was measured as the increment of the contribution score from baseline to follow-up, divided by time interval length in natural logarithmic form. The formula is presented in equation 1 in Figure 5.

The reason for dividing by the number of days between baseline and follow-up in this equation requires explanation. To avoid interfering with the normal operation of the Good Doctor website, our crawler process did not download data very frequently. We spent approximately 3 days collecting all the physicians’ data at one time, as shown in Figure 2. Hence, this time is not precisely equal to 30 days for each physician. Dividing by the number of days, which does not need to be an integer, eliminates this slight estimating bias. Specifically, the physician online contribution is regarded as a daily physician online contribution measure. The other two flow variables related to rewards were also obtained by a similar measurement.

#### Psychological Reward

The number of received thank you letters was used as a proxy for psychological reward. Thank you letters were written by patients to express their thankfulness. The increment of psychological reward is the change in psychological reward measured as the natural logarithm of the increment of thank you letters received from baseline to follow-up divided by the time interval length. The formula is presented in equation 2 in Figure 5.

#### Material Reward

Patients can express gratitude to physicians by purchasing virtual gifts such as virtual flowers, plaques, and pennants on the Good Doctor website. These gifts are converted to cash equivalents and are deposited into the physician’s personal research fund. Thus, the number of token gifts received may be regarded as a proxy for material reward in this study. Specifically, increment in material reward is the change of material reward measured as the natural logarithm of the increment of token gifts received from baseline to follow-up divided by the time interval length. The formula is presented in equation 3 in Figure 5.

#### Control Variables

We employed a number of control variables in this study: past physician online contribution at time *t* [15,16]; mean of doctor review ratings at time *t* [5,26]; a dummy variable for the clinic title, where chief and associate chief physicians were coded as 1 and others were coded as zero [5]; a dummy variable for hospital level, set to 1 if the physician was from a tertiary hospital and zero otherwise [5]; and a dummy variable for city level, set to 1 if the doctor was from Beijing or Shanghai and zero otherwise [5]. In this study, all control variables were stock variables, which represented the online and offline status of physicians at time *t*. The definitions and measurements of all variables are reported in Table 1.

### Statistical Analysis

To examine the research question of whether psychological and material rewards will affect physician online contribution, a multiple linear regression model was constructed as presented in model 1 in Figure 5.

To investigate the concave-down-increasing relationship between rewards and physician online contribution, we further considered a multiple quadratic regression model as presented in model 2 in Figure 5.

To test the curvilinear impact of the square of the increment of psychological (or material) reward, the increment of psychological (or material) reward was mean-centered to reduce the chances of multicollinearity, and multiplied with the original scores [36]. If β_8_ (β_9_) were found to be significantly positive or negative, that result would confirm the nonlinear causal relationship between psychological (or material) reward and physician online contribution.

**Table 1:** Variable definitions and measurements

Variable definitions	Measurements
Increment of physician online contribution	Natural logarithm of the increment of the contribution score from time *t* to *t* +1 divided by time interval length
Increment of psychological reward	Natural logarithm of the increment of thank you letters received from time *t* –1 to *t* divided by the time interval length
Increment of material reward	Natural logarithm of the increment of token gifts received at time *t* –1 and *t* divided by the time interval length
Past online contribution	Natural logarithm of the contribution score of the doctor at time *t*
Doctor review rating	Mean of the overall ratings in user reviews of the doctor at time *t* (on a scale of 1 to 5 with 5 being the highest score)
Clinic title	A dummy variable, coded 1 if the clinic title was chief physician or associate chief physician, 0 otherwise
Hospital level	A dummy variable, coded 1 if the doctor was from the tertiary hospital, 0 otherwise
City level	A dummy variable, coded 1 if the doctor came from Beijing or Shanghai, 0 otherwise

## Results

### Descriptive Statistics

The contribution scores of 40,300 physicians were collected twice, with an interval of approximately 1 month, from July 26, 2017 to August 27, 2017. The increment of all physicians’ contribution scores was 10,609,215, meaning the rate of increase was approximately 2.8%. Thus, the mean increment of the contribution score was approximately 263.2 (SD 701.9) per physician. It was of particular interest to investigate the difference across 10 major specialty areas. Table 2 shows that the contribution scores of the physicians in the specialties of gynecology/obstetrics and pediatrics increased much more than those of others. Specifically, the increments of the contribution scores were mean 413.5 (SD 981.3) and mean 362.2 (SD 822.3) per doctor, respectively. We also observed the increments in the numbers of thank you letters and token gifts in each specialty area. Table 2 indicates that physicians in the surgery and ophthalmology specialties received more thank you letters (ie, 0.85 and 0.80 letters per physician, respectively). The average increments in numbers of token gifts across specialty areas are represented in Table 2. On average, one physician received 1.45 token gifts, but physicians in the pediatrics and gynecology/obstetrics specialties received 1.95 and 1.91 token gifts, respectively.

### Causal Relationship Between Rewards and Physician Online Contribution

Table 3 presents the regression estimation for model 1 with the 40,300 physician sample. We report the standardized regression coefficients, standard errors, *t* values, and *P* values for all variables. The coefficient of determination is relatively high (*R*^2^=.534); that is, the model is able to explain a substantial amount of variance in the dependent variable. The result demonstrates the significant effect of psychological reward on physician online contribution (β_1_=0.192). We also found a positive and significant relationship between material reward and online contribution (β_2_=0.359).

**Table 2:** Mean increments in contribution scores (July 26-August 27, 2017), number of thank you letters (June 25-July 28, 2017), and number of token gifts (June 25-July 28, 2017) by major specialty area.

Specialty	Increment of contribution score Mean (SD^a^)	Increment of number of thank you letters Mean (SD)	Increment of number of token gifts Mean (SD)
Cancer	207.9 (788.8)	0.6 (26.6)	1.5 (143.1)
Gynecology and obstetrics	413.5 (981.3)	0.6 (32.4)	1.9 (231.9)
Internal medicine	210.2 (592.8)	0.5 (26.2)	1.4 (127.3)
Ophthalmology	269.6 (706.7)	0.8 (33.8)	1.3 (129.1)
Oral health	189.2 (485.1)	0.6 (29.0)	0.8 (86.6)
Orthopedics	170.1 (477.8)	0.7 (30.3)	1.0 (122.5)
Pediatrics	362.2 (822.3)	0.7 (34.0)	2.0 (195.1)
Surgery	212.7 (532.3)	0.9 (37.8)	1.5 (152.8)
Traditional Chinese medicine	235.2 (594.3)	0.6 (24.8)	1.1 (143.3)
Others	342.6 (889.7)	0.7 (32.9)	1.5 (179.2)
Total	263.2 (701.9)	0.7 (32.2)	1.5 (160.2)

^a^SD: standard deviation

**Table 3:** Results for the effect of antecedents on online contribution (N=40,300).

Independent variables^a^		Coefficient^b^	SE^c^	*t*_40,292_	*P*
Intercept		1.417	0.011	125.165	<.001
Psychological reward		0.192	0.006	33.112	<.001
Material reward		0.359	0.006	61.827	<.001
**Control variables**					
	Past online contribution	0.450	0.005	89.752	<.001
	Doctor review rating	0.246	0.006	41.823	<.001
	Clinic title	–0.115	0.010	–11.488	<.001
	Hospital level	–0.114	0.012	–9.533	<.001
	City level	–0.149	0.011	–13.084	<.001

^a^Model summary: *R*^2^=.534, *F*_7,40,292_=6588, *P*<.001.

^b^Standardized regression coefficient.

^c^SE: standard error.

For the control variables, the results show that past physician online contribution (β_3_=0.450) and doctor review rating (β_4_=0.246) had positive associations with an increment in physician online contribution, but clinic title (β_5_=–0.115), hospital level (β_6_=–0.114), and city level (β_7_=–0.149) were negatively associated with an increment in physician online contribution. All the estimates were statistically significantly (*P*<.001).

### Quadratic Effect for Impact of Reward on Physician Online Contribution

Table 4 reports the regression analysis results for model 2, including the standardized regression coefficients, standard errors, *t* values, and *P* values for all variables. In comparison to model 1, the coefficient of determination was raised from .534 to .570, meaning the addition of two quadratic variables could improve the original model. All the estimates in Table 4 are statistically significantly (*P*<.001). The characteristics of the coefficients of control variables were very similar to those in the results of model 1. Turning to the effects of psychological and material rewards, the main effects of both rewards were significantly positive (β_1_=0.261 and β_2_=0.688). More importantly, the quadratic effects of rewards were significantly negative (β_8_=–0.015 and β_9_=–0.049). Therefore, the reward did not follow a linear relationship with physician online contribution. In particular, the positive main effect and the negative quadratic effect represent a concave-down-increasing relationship between rewards and physician online contribution.

**Table 4:** Results for the quadratic effect of reward on online contribution (N=40,300).

Independent variables^a^		Coefficient^b^	SE^c^	*t*_40,290_	*P*
Intercept		1.417	0.011	134.762	<.001
**Main effects**					
	Psychological reward	0.261	0.008	34.581	<.001
	Material reward	0.688	0.009	78.670	<.001
**Quadratic effects**					
	(Psychological reward)^2^	–0.015	0.001	–17.549	<.001
	(Material reward)^2^	–0.049	0.001	–49.246	<.001
**Control variables**					
	Past online contribution	0.415	0.005	85.476	<.001
	Doctor review rating	0.189	0.006	32.996	<.001
	Clinic title	–0.099	0.010	–10.296	<.001
	Hospital level	–0.106	0.012	–9.228	<.001
	City level	–0.143	0.010	–13.941	<.001

^a^Model summary: *R*^2^=.570, *F*_9,40,290_=5935, *P*<.001.

^b^Standardized regression coefficient.

^c^SE: standard error.

**Figure 6:**
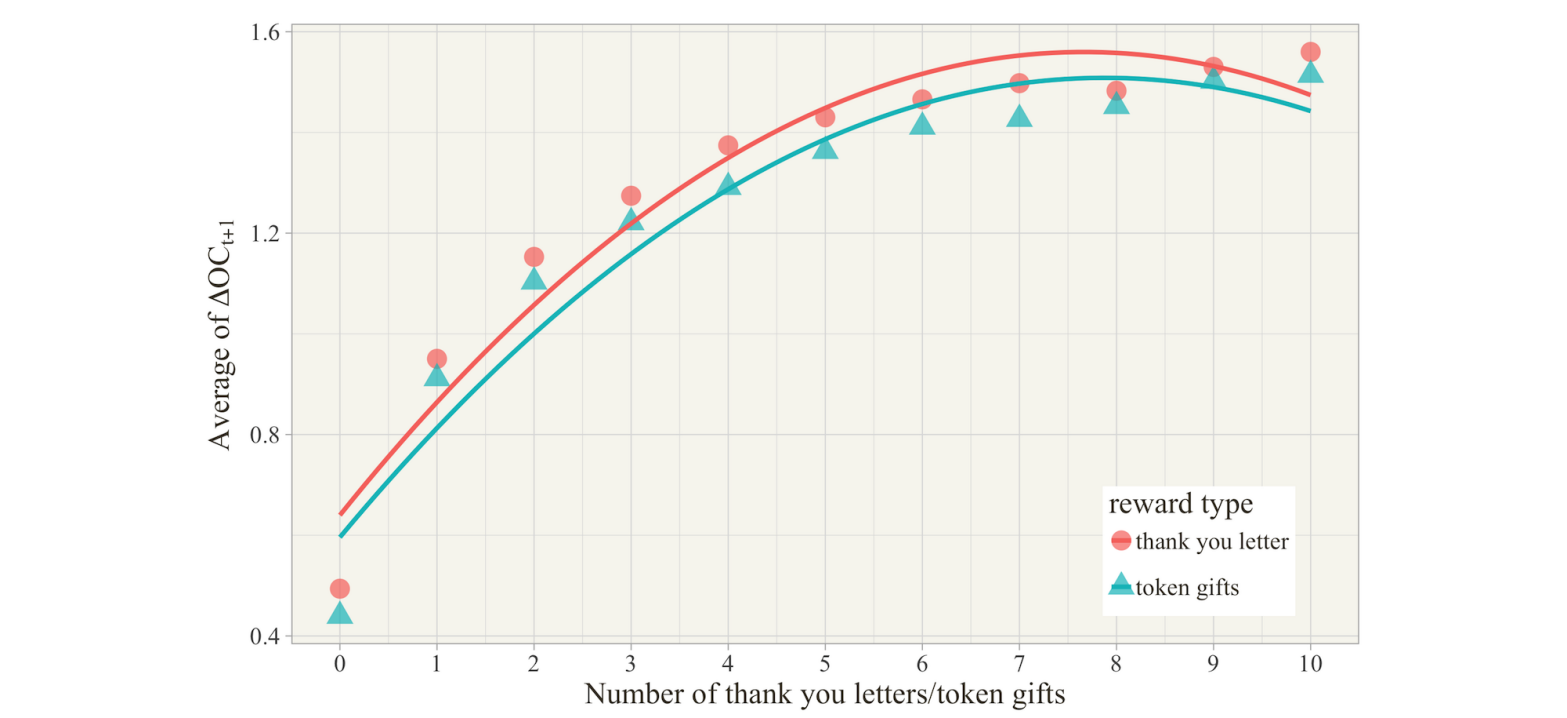
Impact of the number of thank you letters/token gifts on online contribution.

To further understand and verify this relationship, all physicians were grouped by the number of thank you letters (or token gifts) received, and the mean value of the increment of physician online contribution was calculated for each group, with the results depicted in Figure 6. In line with the restriction of the data range, physicians receiving more than 10 thank you letters (or token gifts) were excluded in Figure 6, after which 99.3% (or 97.0%) of all physicians were still included. Both figures clearly illustrate that the main effects of rewards are positive and that the marginal contribution decreases with increasing reward levels.

### Tests for Robustness for the Main and Quadratic Effects of Rewards

Two tests for robustness were performed for this study. We first verified whether the main and quadratic effects of rewards on online contribution were robust for physicians receiving at least one thank you letter or token gift. Specifically, we ignored the relatively inactive physicians, with the result that the sample size was reduced to 16,029. Based on these data, the results of the regression estimations of model 2 are demonstrated in Table 5. The coefficients related to the psychological and material rewards are substantially similar to those presented in Table 4.

**Table 5:** Results for the robustness of the effects of rewards on online contribution for doctors receiving at least one thank you letter or token gift (N=16,029).

Independent variables^a^		Coefficient^b^	SE^c^	*t*_16,019_		*P*	
Intercept		1.759	0.022	81.474		<.001	
**Main effects**							
	Psychological reward	0.143	0.008		17.185		<.001
	Material reward	0.480	0.010		48.730		<.001
**Quadratic effects**							
	(Psychological reward)^2^	–0.005	0.001		–6.097		<.001
	(Material reward)^2^	–0.030	0.001		–27.784		<.001
**Control variables**							
	Past online contribution	0.694	0.011		63.048		<.001
	Doctor review rating	0.079	0.009		8.827		<.001
	Clinic title	–0.184	0.015		–12.342		<.001
	Hospital level	–0.097	0.022		–4.354		<.001
	City level	–0.227	0.015		–14.652		<.001

^a^Model summary: *R*^2^=.531, *F*_9,16,019_=2016, *P*<.001.

^b^Standardized regression coefficient.

^c^SE: standard error.

**Table 6:** Results for the robustness of the effects of rewards on online contribution in the 10 major specialty areas.

Specialty and effects			Psychological reward				Material reward				*R*^2^
			Coefficient^a^		*P*		Coefficient^a^		*P*		
**Surgery**											.592
	Main	0.265		<.001		0.638		<.001			
	Quadratic	–0.022		<.001		–0.048		<.001			
**Internal medicine**											.558
	Main	0.299		<.001		0.778		<.001			
	Quadratic	–0.029		<.001		–0.063		<.001			
**Pediatrics**											.594
	Main	0.303		<.001		0.632		<.001			
	Quadratic	–0.025		<.001		–0.040		<.001			
**Traditional Chinese medicine**											.576
	Main	0.391		<.001		0.806		<.001			
	Quadratic	–0.041		<.001		–0.086		<.001			
**Orthopedics**											.573
	Main	0.281		<.001		0.814		<.001			
	Quadratic	–0.019		<.001		–0.083		<.001			
**Gynecology-obstetrics**											.529
	Main	0.545		<.001		0.585		<.001			
	Quadratic	–0.084		<.001		–0.026		<.001			
**Oral health**											.548
	Main	0.215		<.001		0.883		<.001			
	Quadratic	0.002		0.500		–0.096		<.001			
**Ophthalmology**											.568
	Main	0.355		<.001		0.755		<.001			
	Quadratic	–0.028		<.001		–0.081		<.001			
**Cancer**											.622
	Main	0.102		0.045		0.811		<.001			
	Quadratic	0.020		<.001		–0.069		<.001			
**Others**											.599
	Main	0.286		<.001		0.730		<.001			
	Quadratic	–0.011		<.001		–0.062		<.001			

^a^The standardized regression coefficient related to reward.

More importantly, we further explored the effects of reward on physician online contribution in different specialty areas. Table 6 shows that both the main and quadratic effects of material reward were robust in all specialty areas; these estimates were statistically significant (*P*<.001). For the psychological rewards, the quadratic effects for physicians with oral health and cancer specialties did not have significantly negative coefficients, but the results for other specialties still supported the previous arguments. To sum up, the additional empirical evidence provided here further confirms the robustness of the causal relationship between rewards and physician online contribution.

## Discussion

### Principal Results

The online health care community cannot only reduce medical information asymmetries [37], helping Web users find a good physician or access medical knowledge, but it can also enable patients to communicate directly with physicians online. Thus, the online health care community is a potential solution for the problem of rural-urban health disparities [38], especially in developing countries such as China. Nevertheless, the success of the online health care community depends on whether enough physicians are actively involved in it. We should first realize that our empirical results cannot be used to explain all the physicians’ online and offline contributions to patients, but only their online health care community participation behavior. Our findings showed that, in various specialty areas, the mean levels of physician online contribution were different. Table 2 indicated that the online contribution of physicians in the gynecology/obstetrics and pediatrics specialty areas were much higher than those in other areas. One possible reason is that these physicians have more opportunities to serve patients online or offline. Specifically, pediatrics and obstetrics happen to be a universal event for most people. Most people will not have heart surgery, but a relatively high percentage of couples will decide to have children. In particular, the Chinese people are very concerned about medical issues related to their children, which also creates more opportunities for the physicians to answer patients’ questions. This argument is partially supported by the fact that physicians specializing in gynecology/obstetrics and pediatrics have the largest average numbers of reviews in China [5]. Another possible reason is that the physicians in the gynecology/obstetrics and pediatrics specialty areas are considered to be more people-oriented and compassionate compared with other specialties. Both characteristics may lead them to be more willing to take extra time to help more patients. To sum up, our findings can help people understand the current status of physician online contribution in China, but it should be noted that to criticize any physician for making fewer contributions to the online health care community would be very inappropriate.

We further investigated the factors affecting physician online contribution. We first discussed the results related to the control variables, including past online contribution, doctor review rating, clinic title, hospital level, and city level, which were not easy to manipulate or change in a short time. Both past online contribution and doctor review rating could be regarded as measures of the physician’s past online behavior. Specifically, a physician with a higher past online contribution implies that he or she is more willing to participate in online health care community activities, and a physician with a higher doctor review rating means that he or she has a better reputation in the online health care community. As shown in Tables 3 and 4, a higher past online contribution and doctor review rating led to more online contribution in the next month. These results were consistent with those of prior literature (ie, people’s past contribution was highly correlated with their subsequent contribution) [15,16], and the review rating was a driver for online participation [26]. The other three control variables were related to physicians’ offline status. Chief and associate chief physicians, physicians from tertiary hospitals, and physicians from the cities of Beijing or Shanghai were less involved in the online health care community. Because these control variables were hard for the online health care community managers to manipulate, this study was more concerned with the variables related to incentive mechanisms.

Our research design related to the incentive mechanism has two particular merits. First, based on multiperiod samples, we examined whether physicians’ receiving different levels of reward in the first month would cause their online contribution behavior to be different in the next month. The result showed a clear causal relationship between rewards and physician online contribution, not merely a correlation relationship. Second, we considered both psychological and material rewards, which were measured by the numbers of thank you letters and token gifts, respectively. Although the value of the token gift was not high (¥5-¥100), unlike the thank you letters, they could be converted for economic use. Previous research related to online Q&A communities [18] and open source software development communities [39] found that extrinsic motivation (ie, financial rewards) positively inflated participation contribution, but that intrinsic motivation (ie, self-worth or self-efficacy) might have no significant association with participation contribution. However, our findings showed that both psychological and material rewards could increase physician online contribution significantly. Comparing these two types of rewards, Table 3 shows the standardized regression coefficients of increments of material and psychological rewards were 0.359 and 0.192, respectively, meaning that material reward had a larger effect than psychological reward. Moreover, we examined the multiple quadratic regression model in model 2. The positive main effects (β_1_=0.261, β_2_=0.688) and the negative quadratic effects (β_8_=–0.015, β_9_=–0.049) indicated a concave-down-increasing relationship between rewards and physician online contribution. As shown in Tables 5 and 6, all empirical results were robust for the subset of the sample in which physicians received at least one thank you letter or token gift, and were robust for different specialty areas.

Finally, we make two specific recommendations for online health care community managers based on our findings. First, the means of physician online contribution in various specialty areas are quite different; thus, online health care community managers should make an effort to rebalance the online workload of physicians in different specialties. In particular, Table 2 indicated that online contributions of physicians in gynecology/obstetrics and pediatrics specialty areas were much more than those of others, but the number of those physicians was relatively small, as shown in Figure 3. Thus, online health care community managers should attempt to recruit more physicians within the gynecology/obstetrics and pediatrics specialties. Second, our findings verified the importance of incentive mechanisms in the online health care community. Both psychological and material rewards can make individuals more willing to do something. Because the continued effective operation of the online health care community must rely on physicians’ participation, a feasible incentive mechanism needs to be developed. We propose two guidelines for managers to refer to: (1) material reward is more useful than psychological reward, even if the received economic benefit is very limited, and (2) to maximize the physician online contribution, online health care community managers should avoid excessive concentration of rewards on a small number of physicians. In other words, the appropriate reward level for each physician is enough, since the marginal online contribution decreases with the reward level.

### Limitations and Future Work

We note some limitations and indicate possible future research issues in this section. First, all data were collected from one single online health care community, the Good Doctor website. Although it is the largest and the first online health care community in China, this means our results may only partially reflect the reality of the physician online contribution behavior. Second, because the increment of the contribution score was calculated from July 26 to August 27, 2017, a seasonal bias may exist. For example, physicians with an internal medicine specialty may be busier in the winter than in the summer. In future studies, more interesting results may be found if we can observe the physicians’ online contribution behavior through a cross-season sample. Third, the physician online contribution was measured by the Good Doctor website’s contribution score, which was a quantitative indicator and could not reflect the qualitative value of contributions. For example, if a physician answered five patients’ questions with careful consideration, he or she would still receive a lower contribution score than another physician who responded to more patients’ questions more thoughtlessly. However, to properly measure the quality of contribution is a challenging task.

Two other issues related to physician online contribution can be investigated in future work. First, patients with chronic or acute conditions would come with quite different symptoms and receive different treatment processes [40-42]. In particular, most people with acute illnesses (eg, flu) will soon recover, but chronic health conditions (eg, diabetes) usually cannot be cured, only controlled. The chronic or acute condition might lead to dissimilar physicians’ online contribution behavior. Second, and perhaps more importantly, the physician online contribution must meet different types of social support needs including informational [43] and emotional support [44]. The former can be specified into experience-based information, unconventional information, and medical facts [45]. By contrast, emotional support involves a patient’s emotions or feelings; for instance, physicians need to listen and talk about patients’ concerns in a way that is helpful and reassuring. In terms of mental health, the emotional support may be more important than the informational support. Therefore, it is also worthwhile to explore the type of social support that the physician online contribution provides for patients.

### Conclusions

To summarize, we investigated a novel online health care community in China, which could be regarded as a physician-patient communication platform. If this online health care community functions well, it could alleviate the hospital queuing problem and the problem of inadequate rural medical resources. However, the most important part of this community is the physicians: only when physicians are willing to actively participate does the online health care community have the chance to succeed. Thus, this paper focuses on the topic of physician online contribution.

This study makes several contributions. First, it is the first study to further our understanding of physician online contribution behavior by analyzing a large amount of real data collected from the most popular online health care community in China. Second, our findings can increase the understanding of physician online contribution behavior. We discovered that the averages of online contribution across 10 major specialty areas were different. Specifically, physicians in gynecology/obstetrics and pediatrics specialties are much more involved with the online health care community than others. In addition, physicians with more past online contributions, with higher review ratings, with lower clinic levels, who are not from the tertiary hospitals, and who are not from big cities expend more effort in the online health care community to share their medical knowledge and to help patients. Finally, we found that when physicians received more thank you letters (psychological rewards) or token gifts (material rewards), they were willing to do more the following month, regardless of their specialty areas. The influence of material reward is greater than that of psychological reward. We further found that to enhance online contribution, extreme rewards are marginally less effective than moderate ones. Therefore, our results provide a guide for online health care community managers to design a useful incentive mechanism to improve physician online contribution.

## Abbreviations

SD: standard deviation
SE: standard error
